# GC-MS Based Metabolomics Reveals the Synergistic Mechanism of Gardeniae Fructus-Forsythiae Fructus Herb Pair in Lipopolysaccharide-Induced Acute Lung Injury Mouse Model

**DOI:** 10.1155/2021/8064557

**Published:** 2021-07-27

**Authors:** Wenhui Wu, Huiqing Lin, Ailing Yin, Cunsi Shen, Hongliang Zhou, Majie Wang, Heming Yu, Haidan Wang, Zhihao Zhang, Wei Zhou

**Affiliations:** ^1^State Key Laboratory of Natural Medicines, School of Traditional Chinese Pharmacy, China Pharmaceutical University, Nanjing 210009, China; ^2^Department of Thoracic Surgery, Renmin Hospital of Wuhan University, Wuhan 430060, China; ^3^Nanjing Hospital of Chinese Medicine Affiliated to Nanjing University of Chinese Medicine, Nanjing 210023, China; ^4^Affiliated Hospital of Nanjing University of Chinese Medicine, Nanjing 210029, Jiangsu, China

## Abstract

Compatibility remains among the crucial and significant characteristics of traditional Chinese medicines. The Gardeniae Fructus (FG)-Forsythiae Fructus (FF) herb pair, an epitome of formulations for heat-clearing and detoxification, is extensively used to treat bacterial pneumonia in clinical settings. However, there are few reports on their synergistic effects. This study thus investigated their compatibility by GC-MS based metabolomics using a lipopolysaccharide (LPS)-induced acute lung injury (ALI) mouse model. Differential metabolites were identified by both variable importance in the projection (VIP) > 1 in orthogonal partial least-squares discriminant analysis (OPLS-DA) mode and *P* < 0.05. Results of biochemistry and histopathology indicated that FG-FF herb pair exerted more promising lung protective effect than its individual decoction against the LPS-induced ALI model. From the metabolomics study, 32 differential metabolites in vehicle vs. model groups, 21 differential metabolites in FF vs. model groups, 21 differential metabolites in FG vs. model groups, and 20 differential metabolites in FG-FF herb pair vs. model groups were found. Among them, the levels of 3-hydroxybutyric acid, alanine, isophthalic acid, and terephthalic acid were restored significantly in the FF group, while silanol and cholesterol were restored significantly in the FG group. For FG-FF treatment, the amount of behenic acid, a metabolite with anti-inflammatory properties, was increased, while palmitic acid, a proinflammatory metabolite, was decreased. Meanwhile, the two biomarkers were restored more significantly than that by FG or FF treatment, which indicated that the synergistic effects by FF coupled with FG might be attributed to restoring fatty acids metabolic pathway.

## 1. Introduction

Traditional Chinese medicine (TCM) remains a viable alternative to western medicine for disease prevention and treatment [1]. Herb compatibility represents a key characteristic of TCM [2]. The combination of two herbs, termed an herb pair (known as *Yaodui* or *Duiyao* in Mandarin), is frequently employed for many formulae, a process which leads to either mutual improvement and strengthening or antagonism of pharmacological activity [3]. The Chinese medicine herb pair Gardeniae Fructus (FG) and Forsythiae Fructus (FF) represents a prototype of herbal combinations for heat-clearing (*qing re*) and detoxification (*jie du*). FG has been shown to exhibit diverse pharmacological effects. It is particularly effective in the treatment or management of bacterial infections, hyperlipidemia, influenza A virus infections, diabetes aging [4], and inflammatory conditions of the liver, pancreas, and ligament [5]. Meanwhile, FG has been reported to attenuate esophagitis in rats through antioxidative stress [6]. Geniposide, an active iridoid glycoside component in FG, had protective effects against LPS-induced ALI [7]. FF on the other hand is a known remedy against bacterial, viral infections and melanoma [8], inflammation, and obesity [9]. FF also alleviated liver fibrosis by inhibition of TLR4/MyD88/NF-*κ*B and TAK1 signaling pathway [10]. Shuang-Huang-Lian (SHL), where FF is the main herb of SHL, has effectively attenuated the LPS-induced ALI [11]. In clinic, FG-FF as a combination has widely treated inflammation in bacteria-induced pneumonia [7, 12]. We previously characterized a total of 58 components including the types of iridoid glycosides, crocetin glycosides, monocyclic monoterpene, organic acids, phenylethanoid glycosides, lignans, flavonoids, and saponins in FG-FF decoction using a two-step screening method [13]. However, its mechanism of action remains poorly understood because the various chemical constituents of FG-FF herb pair exhibit diversity in their corresponding treatment targets.

Metabolomics is the whole-scale study to identify and quantify the metabolites, including lipids, amino acids, organic acids, nucleotides, and other small molecules in biological systems by nuclear magnetic resonance (NMR), liquid chromatography-mass spectrometry (LC-MS), or gas chromatography-mass spectrometry (GC-MS) [14]. Comparing genomics, transcriptomics, and proteomics, metabolomics provides the most predictive phenotypic information, which is useful for the comprehensive study of TCM in respect of their efficacies and mechanisms of action [15]. Herein, GC-MS based metabolomics was performed to clarify the anti-inflammatory effects of FG-FF herb pair in lipopolysaccharide (LPS)-induced acute lung injury (ALI) mouse model.

## 2. Experiment

### 2.1. Chemicals and Materials

The ripe fruits of *Gardenia jasminoides* Ellis (Gardeniae Fructus) and *Forsythia Suspense* Thunb. (Forsythiae Fructus) were collected from the Third Affiliated Hospital of Nanjing University of Chinese Medicine (Nanjing, China) and authenticated by Prof. Wu (Department of Pharmacognosy, Nanjing University of Chinese Medicine). TNF-*α* and IL-1*β* commercial ELISA kits were purchased from Biolegend (San Diego, CA, USA). Purified water was obtained using Milli-Q purification system (Millipore, Bedford, MA, USA). Lipopolysaccharide (LPS) was purchased from Sigma-Aldrich (St. Louis, Mo, USA). Dexamethasone (DXMS) was purchased from Xianju Pharmaceutical Co., Ltd. (Zhejiang, China).

### 2.2. Preparation of FG-FF Herb Pair

The accurately weighed raw materials of FG (1000 g) and FF (1000 g) were immersed in 20 L distilled water (1 : 10, *w*/*v*) for 1 h and decocted twice by boiling for 45 min. Then, the two extracts were combined, filtered, and condensed under reduced pressure using a rotary evaporator to obtain a concentration of 1.0 g raw material/mL for FG-FF herb pair stock solution. Meanwhile, the accurately weighed raw materials of FG (2000 g) and FF (2000 g), respectively, were immersed in 20 L distilled water (1 : 10, *w*/*v*) for 1 h and decocted twice by boiling for 45 min. Then, the two extracts were combined, filtered, and condensed to obtain a concentration of 1.0 g raw material/mL for FG and FF.

### 2.3. Animals

Male BALB/c mice (*n* = 60) of weight 18–22 g were purchased for the study. The study was guided by regulations for Care and Use of Laboratory Animals and approved by the Ethics Committee of China Pharmaceutical University. The mice were allowed to adapt to the experimental environment for a minimum of one week. The mice were then randomly grouped into vehicle, model, FG-FF, FF, FG, and DXMS (*n* = 10 per group). The vehicle group and the model group were given PBS by oral administration, the oral dose of 10 g raw material/Kg was used for the FG-FF, FF, and FG groups, and the oral dose of DXMS was 3 mg/Kg. The groups were given continuous administration for 14 days, respectively. As described in a previous study [16], the mice in the model, FG-FF, FF, and FG groups were intranasal instilled 50 *μ*L LPS (0.5 mg/kg b.w) after 1 hour of the last administration, and control group was given 50 *μ*L of PBS by intranasal instillation. The experiment was terminated at six hours after LPS instillation and lung tissues and BALF were harvested and collected thereafter.

### 2.4. Sample Collection

After 6 h of LPS challenge, the animals were euthanized and killed by cardiac puncture. Using a cannula, bronchoalveolar lavage was surgically performed in the trachea. Following instillation of ice-cold PBS (3 × 500 *μ*L) into the airways, recovery of a constant volume of BALF (1400–1450 *μ*L) from each mouse was achieved. The lungs were removed and washed in ice-cold PBS, and the lobule of each lung was used for histopathological analysis.

### 2.5. Histopathological Analysis

Lung specimen of each animal was dehydrated for 12 h in 4% neutral buffered paraformaldehyde. They were then embedded in paraffin wax, sliced into sections of 3 *μ*m thickness, and stained with hematoxylin and eosin (H&E) dye. Examination of lesions in the tissues and infiltrated inflammatory cells was done under a microscope. Using the ranking of scores described by Szarka et al. [17], scores were assigned to each section of lung based upon the degree of endothelial damage, severity of inflammation, percentage of neutrophils in the reaction, number of inflammatory cells in alveoli, and numbers of neutrophils in bronchioles. Depending on the severity of each lesion, the semiquantitative scale is 0–4. The mean score for each animal in each group was calculated.

### 2.6. Measurements of Inflammatory Cytokines

Levels of the inflammatory cytokines, TNF-*α* and IL-1*β*, were determined in the bronchoalveolar lavage fluid (BALF), after centrifuging at 1000 × g for 10 min at 4°C using ELISA kits according to the manufacturer's protocol.

### 2.7. Determination of Inflammatory Cell Count in BALF

The samples (i.e., BALF) were centrifuged at 1000 × g for 10 min at 4°C to precipitate the cells (form cell pellets). These pellets were then resuspended in PBS and the total cell count was determined using a hemocytometer.

### 2.8. GC-MS-Based Lung Metabolomics

#### 2.8.1. Sample Preparation

Accurately weighed 100 mg of lung tissue was homogenized for 5 min in an ice bath by addition of 0.5 mL mixture of methanol and chloroform (3 : 1, *v*/*v*) and 10 *µ*L of myristic acid as internal standard (concentration: 1 mg/ml in methanol), then stored at room temperature for 10 min, and centrifuged at 15 000 × g for 10 min at 4°C. Then 300 *μ*L of supernatants was transferred into a sample vial for vacuum drying at room temperature. The residue was redissolved in 40 *μ*L of a methoxyamine solution (15 mg/mL in pyridine) and vortexed for 1 min. An oximation reaction was performed at 37°C for 1.5 h. 80 *μ*L of BSTFA (containing 1% TMCS) was added to the solution, and the solution was vortexed for 30 s. The sample was kept at 70°C for 1 h and vortexed again for 10 s. The supernatant was transferred to a sample vial for GC-MS analysis.

#### 2.8.2. GC-MS Analysis

The samples were analyzed using an Agilent 7890 chromatograph coupled with a 5977B MS system (Agilent Technologies, Santa Clara, CA, USA). Separation was achieved on a DB-5 ms capillary column coated with 95% dimethyl 5% diphenyl polysiloxane (30 m × 0.25 mm i.d., 0.25 *μ*m film). The initial GC oven temperature was set at 60°C for 1 min, followed by a 10°C/min oven temperature ramp to 325°C, which was maintained for 10 min. The temperature of the inlet, transfer line, and ion source was set to 250, 290, and 250°C, respectively. The injection volume was 1 *μ*L with a split ratio of 1 : 5. Helium was used as the carrier gas with a constant flow rate of 0.87 mL/min. Measurements were made with electron impact ionization (70 eV) in full scan mode (m/z 50−650).

### 2.9. Data Analysis

QC samples were analyzed five times at the beginning of the run and injected once after every 10 injections of the random sequenced samples. The raw data obtained from the GC-MS run were transformed to the m/z data format using MassHunter Workstation Software (Version B.06.00, Agilent Technologies). Data pretreatment including nonlinear retention time alignment, peak discrimination, filtering, alignment, matching, and identification was done using XCMS package (http://metlin.scripps.edu/download/) in R-3.3.3. Relative contents of metabolites in percentage were calculated with Areas of Peak Normalization Method, which was used to eliminate the influence of variations in lung tissue volumes on the level of metabolites. Differential metabolites were tentatively identified by library search (NIST and Fiehn database). The pathway analysis for metabolites was performed by KEGG using MetaboAnalyst software. All the pretreated data were normalized by LOESS before multivariate analysis. Mann–Whitney test was used to analyze the statistical significance of the results.*P* values less than 0.05 were considered to be of statistical significance. The heatmap was obtained by cluster analysis. The dissimilarity test among groups was conducted by the vegan package in R-3.4.3. OPLS-DA and other analyses were performed by R-3.4.3. Statistical analysis was performed using SPSS software version 19.0 (IBM Corp., Armonk, New York).

## 3. Results

### 3.1. Effect of FG-FF Herb Pair on Lipopolysaccharide-Induced Acute Lung Injury Mouse Model

**Figure 1:**
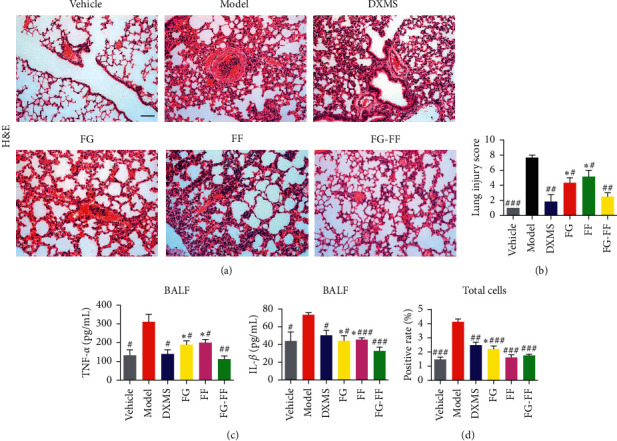
Effect of Gardeniae Fructus (FG)-Forsythiae Fructus (FF) herb pair on lipopolysaccharide (LPS)-induced acute lung injury (ALI) mouse model. (a) Representative micrographs of lung tissue sections stained with H&E. (b) Lung injury score based on the degree of inflammation. (c) The concentration of TNF-*α* and IL-1*β* in the bronchoalveolar lavage fluid. (d) The total cell count in the bronchoalveolar lavage fluid. ^#^*P* < 0.05, ^##^*P* < 0.01, and ^###^*P* < 0.001 vs. model group, ^*∗*^*P* < 0.05, ^*∗∗*^*P* < 0.01, and ^*∗∗∗*^*P* < 0.01 vs. FG-FF group.

The cytokines (TNF-*α* and IL-1*β*) and inflammatory cell counts in BALF, as well as the lung histopathological scores, were all markedly elevated after LPS stimulation, but almost restored to normalcy after DXMS treatment, indicating that the LPS-induced ALI model was successfully established in the mice (Figure 1). We then found that the TNF-*α* and IL-1*β*, as well as the counts of total inflammatory cells in BALF and lung injury scores after FF, FG, and FG-FF herb pair treatment, respectively, were significantly alleviated in comparison of model groups, and the effect of combination treatment was better than that of FF or FG group (Figure 1). The biochemical and histopathological tests indicate that the treatment of LPS-induced ALI in mice after FG-FF herb pair showed a promising synergistic effect.

### 3.2. GC-MS-Based Metabolomics Reveals Underlying Mechanisms of FG-FF Herb Pair in Lipopolysaccharide-Induced Acute Lung Injury Mouse Model

**Figure 2:**
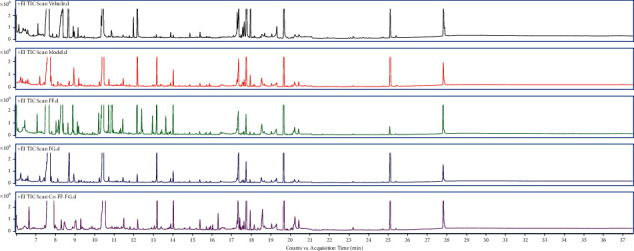
The total ion chromatograms (TICs) of lung samples for vehicle, model, Forsythiae Fructus (FG), Forsythiae Fructus (FF), and Gardeniae Fructus (FG)-Forsythiae Fructus (FF) groups.

**Figure 3:**
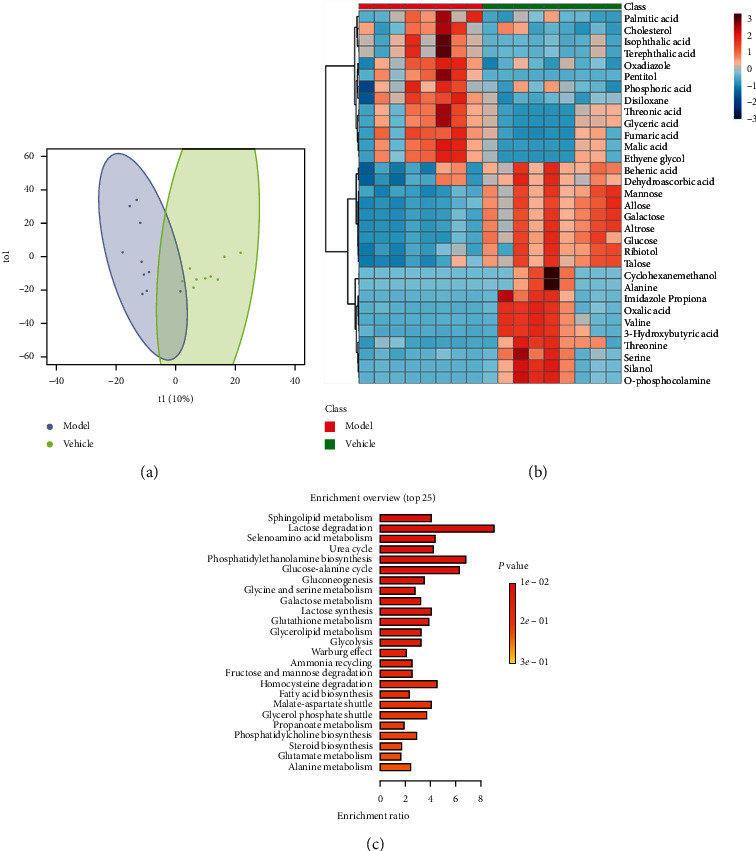
Lung metabolomics based on GC-MS for comparison of vehicle and model groups. (a) OPLS-DA scores plot of vehicle vs. model. (b) Heatmap of differential metabolites. (c) KEGG analysis of differential metabolites.

**Figure 4:**
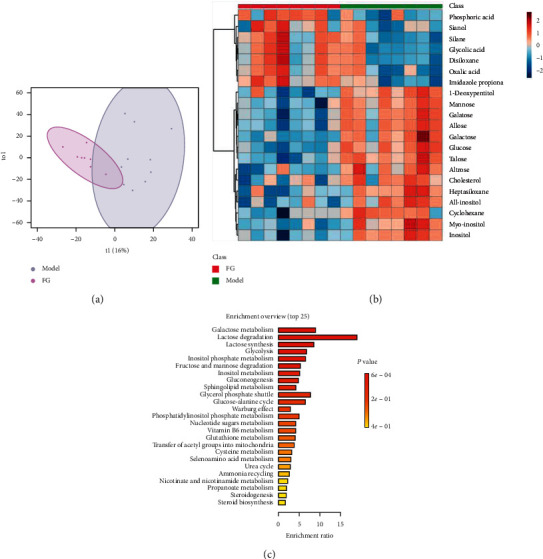
Lung metabolomics based on GC-MS for comparison of model and Gardeniae Fructus (FG) groups. (a) OPLS-DA scores plot of model vs. FG groups. (b) Heatmap of differential metabolites. (c) KEGG analysis of differential metabolites.

**Figure 5:**
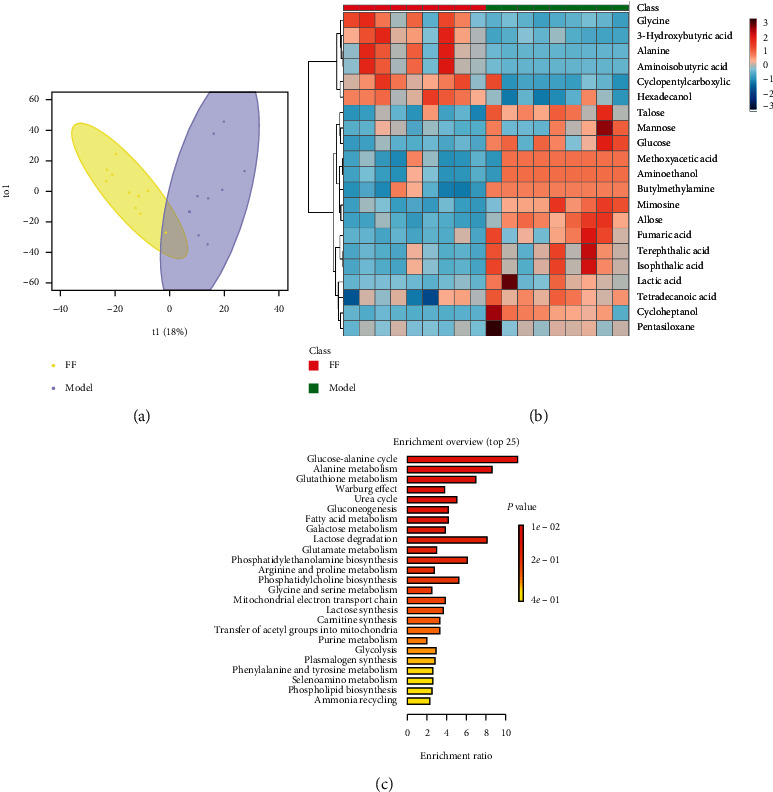
Lung metabolomics based on GC-MS for comparison of model and Forsythiae Fructus (FF) groups. (a) OPLS-DA scores plot of model vs. FF groups. (b) Heatmap of differential metabolites. (c) KEGG analysis of differential metabolites.

**Figure 6:**
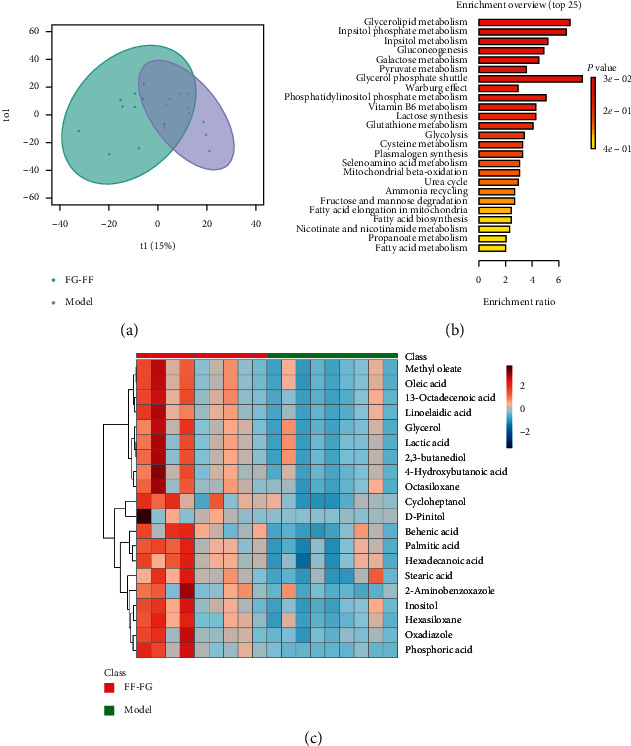
Lung metabolomics based on GC-MS for comparison of model and Gardeniae Fructus (FG)-Forsythiae Fructus (FF) herb pair groups. (a) OPLS-DA scores plot of model vs. FG-FF groups. (b) Heatmap of differential metabolites. (c) KEGG analysis of differential metabolites.

**Figure 7:**
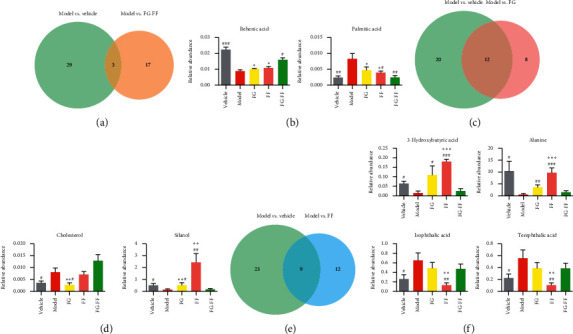
Restored differential metabolites after oral administration of Gardeniae Fructus (FG)-Forsythiae Fructus (FF) herb pair. (a) Venn diagram of differential metabolites between vehicle vs. model and model vs. Gardeniae Fructus (FG)-Forsythiae Fructus (FF) herb pair groups. (b) Relative abundance for restored differential metabolites between vehicle vs. model and model vs. FG-FF groups. (c) Venn diagram of differential metabolites between vehicle vs. model and model vs. FG groups. (d) Relative abundance for restored differential metabolites between vehicle vs. model and model vs. FG groups. (e) Venn diagram of differential metabolites between vehicle vs. model and model vs. Forsythiae Fructus (FF) groups. (f) Relative abundance for restored differential metabolites between vehicle vs. model and model vs. FF groups. ^#^*P* < 0.05, ^##^*P* < 0.01, and ^###^*P* < 0.001 vs. model group, ^*∗*^*P* < 0.05, ^*∗∗*^*P* < 0.01, and ^*∗∗∗*^*P* < 0.01 vs. FG-FF group.

Metabolic profilings of lung tissue samples (vehicle, model, FF, FG, and FG-FF groups) were acquired using GC-MS. After peak detection and alignment of all total ion chromatograms (TICs) (Figure 2), a total of 1568 features were enrolled in the final data set for the statistical analysis. To evaluate the alterations of metabolome in each group, a supervised OPLS-DA was performed using data from model vs. vehicle groups, model vs. FF groups, and model vs. FG groups, as well as from model group vs. FG-FF groups. OPLS-DA score plots could readily be divided into two clusters (Figure 3(a)), indicating that lung metabolic states in LPS-induced ALI mice model were significantly changed in relation to the vehicle group. Also, a clear distinction in the metabolomes between FF, FG, and FG-FF vs. model groups, respectively, was observed (Figures 4(a), 5(a), and 6(a)), suggesting the metabolic states of ALI mice after oral administration of FF, FG, or its combination groups were significantly different from the ALI group. The OPLS-DA model parameters of *R*^2^*X*, *R*^2^*Y*, and *Q*^2^ for each comparison were as follows: model vs. vehicle (*R*^2^*X* = 0.773, *R*^2^*Y* = 0.808, *Q*^2^ = 0.856); FF vs. model (*R*^2^*X* = 0.776, *R*^2^*Y* = 0.839, *Q*^2^ = 0.688); FG vs. model (*R*^2^*X* = 0.75, *R*^2^*Y* = 0.792, *Q*^2^ = 0.322); FG-FF vs. model (*R*^2^*X* = 0.731, *R*^2^*Y* = 0.694, *Q*^2^ = 0.617). Differentially expressed metabolites were selected using VIP (VIP > 1) and *P* < 0.05 (Tables 1–4). To further understand the metabolic changes, heatmaps were used to visualize the change of metabolites in each group. For model vs. vehicle groups, 32 differential metabolites were found. In comparison to vehicle group, the pentitol, glyceric acid, and ethylene glycol were significantly improved, but altrose, glucose, and galactose were significantly decreased (Figure 3(b)). The differential metabolites were involved in sphingolipid metabolism, lactose degradation, selenoamino acid metabolism, etc. (Figure 3(c)). For FG vs. model groups, 21 differential metabolites were found. The imidazole propionate, silanol, and phosphoric acid were significantly increased, but cyclohexane, allo-inositol, and mannose were significantly decreased compared with the model group and were involved in galactose metabolism, lactose degradation, and lactose synthesis (Figures 4(b) and 4(c)). For FF vs. model groups, we found 21 differential metabolites. Compared with the model group, the alanine, aminoisobutyric acid, and hydroxybutyric acid were significantly increased, but mimosine, terephthalic acid, and cycloheptanol were significantly decreased and were involved in metabolic pathways of glucose-alanine cycle, alanine metabolism, and glutathione metabolism (Figures 5(b) and 5(c)). For FG-FF herb pair vs. model groups, we showed 20 differential metabolites, and the phosphoric acid, D-pinitol, and behenic acid in FG-FF group were significantly higher than those in model group. The disturbed metabolic pathways were involved in glycerolipid metabolism, inpsitol phosphate metabolism, and inpsitol metabolism (Figure 6(b) and 6(c)). Importantly, we found 3 common differential metabolites from model vs. vehicle and FG-FF herb pair vs. model, 9 common differential metabolites from model vs. vehicle and FF vs. model, and 12 common differential metabolites from model vs. vehicle and FG vs. model. The levels of behenic acid and palmitic acid were significantly restored in the FG-FF group; 3-hydroxybutyric acid, alanine, isophthalic acid, and terephthalic acid were restored in the FF group, and silanol and cholesterol were restored in the FG group (Figure 7).

## 4. Discussion

As an important part of TCM, compatibility remains among the crucial and significant characteristics of traditional Chinese medicines; and herb pair is considered the most fundamental and simplest form. The FG-FF herb pair, an epitome of formulations for heat-clearing and detoxification, is extensively used to treat bacterial pneumonia in clinics. However, there are few reports on the mechanisms that underlie its pharmacological effects. GC-MS based metabolomics for identification of perturbed metabolic pathways and networks that can be restored by the administration of FG-FF herb pair seems a fruitful approach. It can also serve as a starting point for further molecular mechanistic studies.

LPS, a major component of the outer membranes in Gram-negative bacteria, has been recognized as the most important pathogen in pulmonary inflammation that leads to ALI [18]. In previous studies, the concentrations of TNF-*α* and IL-1*β* in BALF were determined, and the peak value was reached 6 h after LPS administration [19]. Currently, we found that TNF-*α*, IL-1*β*, and the counts of total inflammatory cells in BALF and lung injury scores after the administration of FF, FG, and FG-FF herb pair, respectively, were significantly alleviated compared with model groups, and the effect of FG-FF was better than that of FF or FG alone. Previous studies have shown that Shuang-Huang-Lian (composition of Forsythiae Fructus, Scutellariae Radix, and Lonicerae Japonicae Flos) can alleviate ALI by inhibition of inflammatory and oxidative effects [11]. Geniposide, the major ingredient of FG, can protect against ALI through inhibition of NF-*κ*B and MAPK signaling pathways [7]. Our research showed that FF, FG, and FG-FF could inhibit inflammation and readjust disturbed metabolic pathway to relieve ALI.

A metabolomics study showed that the induction of ALI may be related to the sphingolipid metabolism, retinol metabolism, and tryptophan metabolism in LC-MS [20]. In our study, GC-MS based lung metabolomics was performed to analyze biomarkers and restored metabolic pathways. We found that 3-hydroxybutyric acid, alanine, isophthalic acid, and terephthalic acid were restored in the FF group. These metabolites were involved in selenocompound metabolism, alanine, aspartate, and glutamate metabolism and aminoacyl-tRNA biosynthesis. 3-Hydroxybutyric acid, an endogenic NLRP3 inflammasome and histone deacetylases inhibitor, activates the anti-inflammatory GPR109A signaling and attenuates stress-induced behavioral and inflammatory responses [21–23]; it can also upregulate FOXO1 and HO-1 by reducing inflammatory responses and apoptotic cell death via the downregulation of NF-*κ*B and NLRP3 inflammasome [24]. The production of 3-hydroxybutyric acid during excess alcohol consumption has an anti-inflammatory and hepatic-protective role through an Hcar2 dependent pathway [25]. Collectively, 3-hydroxybutyric acid as an anti-inflammatory metabolite was upregulated in FF compared to the FG and FG-FF groups. Silanol and cholesterol were also restored in the FG group. The disturbed metabolic pathways included steroid biosynthesis, primary bile acid biosynthesis, and steroid hormone biosynthesis. Like QZJFD (Qingzao Jiufei Decoction), it regulated BA (bile acid), AA (arachidonic acid), and FAs (fatty acids) metabolism to ameliorated ALI [26]. These pathways were identified as potential ALI biomarkers in rats [27]. For the herb pair group, behenic acid (22 : 0) and palmitic acid (16 : 0) were 0.36-fold downregulated and 2.51-fold upregulated in model compared to the vehicle group, but administration of FG-FF significantly attenuated the downregulation of behenic acid and upregulation of palmitic acid compared to the model group. These metabolites were involved in the biosynthesis of unsaturated fatty acids, fatty acid elongation, fatty acid degradation, and fatty acid biosynthesis. The differentially regulated lipids were reported to be the potential biomarkers for ALI [28]. PSG (Ganoderma atrum polysaccharide) could attenuate ALI by regulating SCFAs (saturated fatty acids) [29]. It was reported that behenic acid identifies as a natural pancreatic lipase inhibitor. Lowering triglyceride absorption by inhibiting pancreatic lipase in the presence of behenic acid reduces LPS absorption, then resulting in the reductions of inflammatory signaling, NF-*κ*B activation, and cytokine gene expression. Thus, it has a reduced ability to induce postprandial inflammation [30]. We also found that behenic acid is positively associated with disease severity after H7N9 and COVID-19 infection in humans [31]. Additionally, palmitic acid is a saturated fatty acid. It was found that blood concentration is elevated in obese patients. Palmitic acid is not only a TLR agonist, but also converted into phospholipids, diacylglycerol, and ceramides that trigger the activation of various signaling pathways, such as LPS-mediated TLR4. In particular, metabolic products of palmitic acid affect the activation of various PKCs and ER stress that cause an increase in ROS generation [32]. In summary, behenic acid elicits anti-inflammatory effects, while palmitic acid is involved in inflammation. Importantly, we found that the level of behenic acid and palmitic acid restored by FG-FF was better significantly than that by FG or FF treatment, which indicated that the synergistic effects might be attributed to the restored fatty acids metabolism pathway more significantly than that by individual treatments.

## 5. Conclusion

Our findings indicate that the disturbed fatty acids metabolic pathway restored more significantly by administration of FG-FF herb pair than that by FG or FF treatments might be the synergistic protective mechanism on LPS-induced ALI mice model using GC-MS based metabolomics. This study not only strengthens the rationale behind the use of traditional Chinese prescriptions containing FG and FF, but also explains the compatibility mechanism of the herb pair.

## Figures and Tables

**Table 1:** The differential metabolites for comparison of vehicle vs. model.

Primary ID	Model vs. vehicle				
	*m*/*z*	RT (min)	FC (model/vehicle)	^b^VIP	*P*
Cyclohexanemethanol	100	6.00	0.31	1.55	3.5 × 10^∧−2^
Alanine	146	6.01	0.29	1.56	3.5 × 10^∧−2^
Oxalic acid	116	8.31	0.5	1.59	2.9 × 10^∧−2^
Valine	91	8.32	0.5	1.6	1.9 × 10^∧−2^
3-Hydroxybutyric acid	99	8.32	0.49	1.62	4.3 × 10^∧−2^
Imidazole propionate	115	8.32	0.5	1.61	3.5 × 10^∧−2^
Phosphoric acid	214	10.41	1.75	1.19	2.9 × 10^∧−2^
Oxadiazole	256	10.43	2.75	1.77	3.0 × 10^∧−3^
Disiloxane	287	10.43	2.1	2.17	2.0 × 10^∧−3^
Silanol	174	10.87	0.29	2.07	9.0 × 10^∧−3^
O-phosphocolamine	276	10.87	0.28	2.06	1.1 × 10^∧−2^
Threonine	158	10.87	0.39	2.14	1.9 × 10^∧−2^
Serine	144	10.88	0.37	2.13	1.9 × 10^∧−2^
Fumaric acid	143	11.43	2.63	2.06	1.5 × 10^∧−2^
Malic acid	134	13.17	2.37	1.85	4.3 × 10^∧−2^
Ethylene glycol	171	13.17	3.03	2.02	2.3 × 10^∧−2^
Threonic acid	217	14.01	2.65	1.97	2.9 × 10^∧−2^
Glyceric acid	277	14.01	3.3	2.29	3 × 10^∧−3^
Pentitol	231	14.59	10.2	1.92	5.0 × 10^∧−3^
Ribitol	142	17.27	0.25	1.36	2.0 × 10^∧−4^
Behenic acid	127	17.28	0.36	1.27	2.0 × 10^∧−3^
Dehydroascorbic acid	143	17.31	0.51	1.43	4.0 × 10^∧−3^
Glucose	102	17.76	0.07	1.67	1.0 × 10^∧−5^
Allose	110	17.76	0.1	1.32	1.0 × 10^∧−5^
Talose	111	17.77	0.15	1.33	4.3 × 10^∧−5^
Mannose	107	17.77	0.12	1.32	1.0 × 10^∧−5^
Galactose	103	17.77	0.07	1.43	1.1 × 10^∧−5^
Altrose	126	17.77	0.09	1.4	1.0 × 10^∧−5^
Palmitic acid	388	19.23	2.51	1.46	1.5 × 10^∧−2^
Isophthalic acid	121	25.08	2.49	1.71	2.3 × 10^∧−2^
Terephthalic acid	113	25.08	2.52	1.72	2.3 × 10^∧−2^
Cholesterol	343	27.80	2.02	1.51	2.1 × 10^∧−2^

**Table 2:** The differential metabolites for comparison of vehicle vs. Gardeniae Fructus (FG) groups.

Primary ID	FG vs. model				
	*m*/*z*	RT (min)	FC (FG/model)	VIP	*P*
Silanol	174	10.31	6.9	1.20	4.3 × 10^∧−2^
Silane	58	8.33	4.6	1.19	8.5 × 10^∧−3^
Phosphoric acid	184	10.43	6.1	1.50	1.1 × 10^∧−2^
Oxalic acid	119	8.32	5.8	1.11	2.1 × 10^∧−2^
Myo inositol	139	19.66	0.4	1.30	2.6 × 10^∧−2^
Imidazole propionate	102	8.38	11.6	1.15	1.3 × 10^∧−3^
Heptasiloxane	382	28.54	0.3	1.22	4.3 × 10^∧−2^
Glycolic acid	71	8.32	5.8	1.12	6.2 × 10^∧−3^
Mannose	279	17.77	0.1	1.95	5.5 × 10^∧−4^
Disiloxane	60	8.32	5.7	1.12	1.2 × 10^∧−2^
Glucose	256	17.77	0.1	1.91	4.4 × 10^∧−3^
Galactose	268	17.78	0.1	1.85	8.5 × 10^∧−3^
Inositol	408	19.67	0.2	1.30	3.4 × 10^∧−2^
Allose	198	17.76	0.2	1.97	2.1 × 10^∧−2^
Altrose	98	17.77	0.5	1.53	4.3 × 10^∧−2^
Galactose	154	17.77	0.4	1.79	2.1 × 10^∧−2^
Cyclohexane	237	6.29	0.1	1.27	8.7 × 10^∧−4^
Cholesterol	343	27.80	0.4	1.41	1.4 × 10^∧−2^
Allo-inositol	310	19.67	0.1	1.12	1.6 × 10^∧−2^
Deoxypentitol	231	13.65	0.3	1.60	3.4 × 10^∧−2^
Talose	241	17.77	0.3	1.85	4.3 × 10^∧−3^

**Table 3:** The differential metabolites for comparison of vehicle vs. Forsythiae Fructus (FF) groups.

Primary ID	FF vs. model				
	*m*/*z*	RT (min)	FC (FF/model)	VIP	*P*
Methoxyacetic acid	89	6.00	0.4	1.48	2.0 × 10^∧−3^
Aminoethanol	101	6.00	0.3	1.60	2.0 × 10^∧−3^
Butylmethylamine	60	6.00	0.4	1.88	9 × 10^∧−4^
Cycloheptanol	237	6.29	0.1	1.82	4 × 10^∧−4^
Cyclopentylcarboxylic acid	163	6.31	2.5	1.38	2.0 × 10^∧−3^
Lactic acid	78	7.56	0.4	1.29	7.0 × 10^∧−4^
Hydroxybutyric acid	86	8.88	4.2	1.93	2.8 × 10^∧−3^
Pentasiloxane	266	8.92	0.4	1.21	2.0 × 10^∧−3^
Mimosine	392	9.33	0.2	1.65	1.0 × 10^∧−3^
Glycine	142	10.75	3.6	1.91	3.8 × 10^∧−3^
Alanine	158	10.87	35.7	1.78	2.0 × 10^∧−4^
Aminoisobutyric acid	100	10.88	35.0	1.73	3.2 × 10^∧−4^
Fumaric acid	361	11.45	0.5	1.54	2.1 × 10^∧−3^
Tetradecanoic acid	69	17.33	0.8	1.20	2.1 × 10^∧−3^
Allose	131	17.76	0.3	1.83	2.1 × 10^∧−4^
Mannose	162	17.76	0.3	1.45	3.9 × 10^∧−3^
Talose	99	17.76	0.5	1.90	4.8 × 10^∧−4^
Glucose	107	17.77	0.4	1.83	1.5 × 10^∧−3^
Hexadecanol	299	18.46	2.1	1.69	1.3 × 10^∧−4^
Terephthalic acid	84	25.08	0.2	1.68	1.5 × 10^∧−3^
Isophthalic acid	55	25.08	0.2	1.70	2.8 × 10^∧−3^

**Table 4:** The differential metabolites for comparison of vehicle vs. Forsythiae Fructus (FF)-Gardeniae Fructus (FG) herb pair groups.

Primary ID	FF-FG herb pair vs. model				
	*m*/*z*	RT (min)	FC (FF-FG herb pair/model)	VIP	*P*
Cycloheptanol	140	6.12	2.5	1.56	1.5 × 10^∧−2^
Lactic acid	102	7.62	2.1	1.13	2.9 × 10^∧−2^
2,3-Butanediol	115	7.62	2.1	1.12	2.8 × 10^∧−2^
4-Hydroxybutanoic acid	233	9.90	2.6	1.29	2.9 × 10^∧−2^
Glycerol	159	10.39	1.9	1.24	4.3 × 10^∧−2^
Oxadiazole	287	10.43	2.6	1.71	1.1 × 10^∧−3^
Phosphoric acid	184	10.43	17.4	1.93	7.5 × 10^∧−5^
2-Aminobenzoxazole	614	13.88	2.0	1.55	1.5 × 10^∧−2^
D-pinitol	449	17.77	12.9	1.17	9.9 × 10^∧−3^
Palmitic acid	117	19.26	1.6	1.74	3.5 × 10^∧−2^
Behenic acid	315	19.27	2.7	1.84	1.9 × 10^∧−3^
Hexadecanoic acid	132	19.27	1.5	1.67	3.5 × 10^∧−2^
Inositol	329	19.66	2.4	1.64	3.5 × 10^∧−2^
Methyl oleate	68	20.20	2.2	1.20	4.3 × 10^∧−2^
Oleic acid	108	20.20	2.3	1.25	4.3 × 10^∧−2^
13-Octadecenoic acid	137	20.22	2.6	1.44	2.3 × 10^∧−2^
Linoelaidic acid	282	20.23	2.4	1.28	2.9 × 10^∧−2^
Stearic acid	356	21.04	1.7	1.34	3.5 × 10^∧−2^
Octasiloxane	430	27.74	2.0	1.18	3.6 × 10^∧−2^
Hexasiloxane	501	30.94	2.6	1.60	4.3 × 10^∧−2^

## Data Availability

The data from this study are available from the authors upon request.
